# Bayesian network-based missing mechanism identification (BN-MMI) method in medical research

**DOI:** 10.1186/s12911-021-01677-6

**Published:** 2021-11-12

**Authors:** Tingyan Yue, Tao Zhang

**Affiliations:** 1 West China Second University Hospital, Sichuan University, Chengdu, China; 2 West China School of Public Health and West China Fourth Hospital, Sichuan University, Chengdu, China

## Abstract

**Background:**

Traditional approaches to identify missing mechanisms are usually based on the hypothesis test and confronted with both theoretical and practical challenges. It has been proved that the Bayesian network is powerful in integrating, analyzing and visualizing information, and some previous researches have verified the promising features of Bayesian network to deal with the aforementioned challenges in missing mechanism identification. Based on the above reasons, this paper explores the application of Bayesian network to the identification of missing mechanisms for the first time, and proposes a new method, the Bayesian network-based missing mechanism identification (BN-MMI) method, to identify missing mechanism in medical research.

**Methods:**

The procedure of BN-MMI method consists three easy-to-implement steps: estimating the missing data structure by the Bayesian network; assessing the credibility of the estimated missing data structure; and identifying the missing mechanism from the estimated missing data structure. The BN-MMI method is verified by simulation research and empirical research.

**Results:**

The simulation study verified the *validity*, *consistency* and *robustness* of BN-MMI method, and indicated its outperformance in contrast to the traditional logistic regression method. In addition, the empirical study illustrated the applicability of BN-MMI method in the real world by an example of medical record data.

**Conclusions:**

It was confirmed that the BN-MMI method itself, together with human knowledge and expertise, could identify the missing mechanisms according to the probabilistic dependence/independence relations among variables of interest. At the same time, our research shed light upon the potential application of BN-MMI method to a broader range of missing data issues in medical studies.

## Introduction

The missing data issues are common in medical researches [1–5]. According to Little and Rubin [6], the mechanisms that lead to data missingness could be summarized as the following three categories: (1) Missing completely at random (MCAR), which suggests the missingness of data elements is not associated with other variables. (2) Missing at random (MAR), which means the missingness of data elements only relate to other observed variables in data set. In other words, the missing data element could be inferred from other observed variables. (3) Missing not at random (MNAR), which suggests the missingness of data elements is related to the missing values. At the same time, the MNAR mechanism is difficult to identify because the real value of missing value is unknown [7].

From the methodological side, the missing mechanisms generate the fundamental basis of method selection for solving missing data. That is, almost all the methods dealing with missing data are based on certain assumptions of the missing mechanisms [8]. For example, the widely-used complete case analysis method (i.e., to directly delete the observations with missing data elements) assumes MCAR mechanism, while the multiple imputation method (i.e., replacing a missing data element with multiple substituted values derived from some certain statistical model) assumes the missing mechanism to be MAR. More importantly, missing mechanism is crucial to the validity of results. In a 5-year follow up of a randomized controlled trial analyzing the difference in the incidence of urinary incontinence between subtotal with total abdominal hysterectomies, multiple imputation method was carried out to deal with missing data, and p-value just changed from 0.026 to 0.052 after such implementation [9]. Thus, we could say that some potential confusions and biases are likely to occur without careful consideration on missing mechanism.

So far, a variety of methods for missing mechanism identification have been proposed. Curran [10] discussed two kinds of methods to identify data missing mechanisms. The first kind is to collect information about why the quality of life questionnaires is not complete, so as to determine the reasons for the lack of the data, and then make judgement about the mechanism for missing data. The second kind is to model the missing data mechanism, and perform hypothesis testing to judge the missing mechanism using logistic regression. Chen [11] proposed a test method for MCAR based on the likelihood ratio test, so as to determine whether the generalized estimation equation should be adjusted to correct the bias introduced by a missing data mechanism that is not MCAR. At the same time, there are corresponding software packages to realize the identification of data missing mechanism. For example, Jamshidian [12] introduced R software package MissMech, which can identify the missing mechanism using a function called TestMCARNormality. The logistic regression method is one of the most widely used methods. It sets the missing indicator (i.e., a dichotomous variable representing whether a given variable has been observed) as dependent variable, and other prior-selected variables as independent ones. Then the missing mechanism is identified according to the hypothesis test of the logistic regression coefficients, that is, if some of the regression coefficients are statistically significant, then the missing mechanism is identified as MAR [13].

Although the methods to identify missing mechanisms are relatively abundant in terms of quantity, there are still great challenges both in theory and in practice. The theoretical challenges arise from hypothesis tests. Most of the current missing mechanism identification methods are essentially based on the hypothesis test [14]. As a consequence, if *P* < 0.05 under the null hypothesis that “the missing mechanism is MCAR”, then one would reject the null hypothesis and believe the missing mechanism is not MCAR; otherwise, if *P* > 0.05, one cannot reject the null hypothesis. However, according to the theory of hypothesis test, “one cannot reject to believe the null hypothesis” is essentially not equivalent to “one accepts the null hypothesis”. Therefore, it is logistically problematic and risky to conclude the MCAR mechanism simply due to p-value. In addition, the sample size requirement of hypothesis test also poses a practical challenge in medical studies [15], where it takes a long time for data collection and the costs are quite high, let alone the data missingness makes it even harder. Another practical challenge comes from the requirement for high methodological proficiency in method implementation as well as result explanation, which throws obstacles for non-statisticians and thus blocks the application of such methods.

In order to deal with those challenges, this paper proposes a new method, the Bayesian network-based missing mechanism identification (BN-MMI) method. As a graphical model to identify the associations among multiple variables, the Bayesian network uses the nodes to represent variables, and the arcs to represent the relations among them. It has been proved that the Bayesian network is powerful in integrating, analyzing and visualizing information [16]. Since the theory of Bayesian network does not rely on hypothesis test, it is able to avoid the aforementioned dilemma of interpreting p-value. In addition, the work of Zou and Feng has already shown that the Bayesian network could perform well even when the sample size is small [17]. Furthermore, as a graphical model, the Bayesian network offers a visual presentation that is easy to understand and utilize. Besides, it should be noted that there are some successful applications of Bayesian network to predict missing values in clinical set [18]. These work validated the promising features of Bayesian network. However, it should be noted that identifying missing mechanism and predicting missing values are two different issues, and almost all the methods dealing with missing data are based on certain assumptions of the missing mechanisms [8]. Therefore, it is still worthwhile to evaluate the performance of Bayesian network in terms of identifying missing mechanism. In this paper, Bayesian network is applied to missing mechanism identification for the first time.

Considering this is a preliminary study of the application of Bayesian network to missing mechanism identification, this paper puts main focus on the issue of univariate other than multivariate missingness, where the former defines only one variable has missing data element(s) and the latter indicates more than one variable has missing data elements [19]. Such consideration is not only due to the convenience of implementation and explanation, but also the current fact that the missing mechanism identification methods for the univariate missingness outperform those for the multivariate missingness, in terms of quantity and maturity. This makes the univariate missingness a good starting point for further research.

The remainder of this paper is organized as follows. In "The framework of BN-MMI method" section we present the framework of BN-MMI method. "Simulation study" section verifies the properties of our method through simulation study. We also illustrate the application of our method with a real data example in section "Empirical study". Finally, "Discussion" section ends the paper with discussion.

## The framework of BN-MMI method

**Fig. 1:**
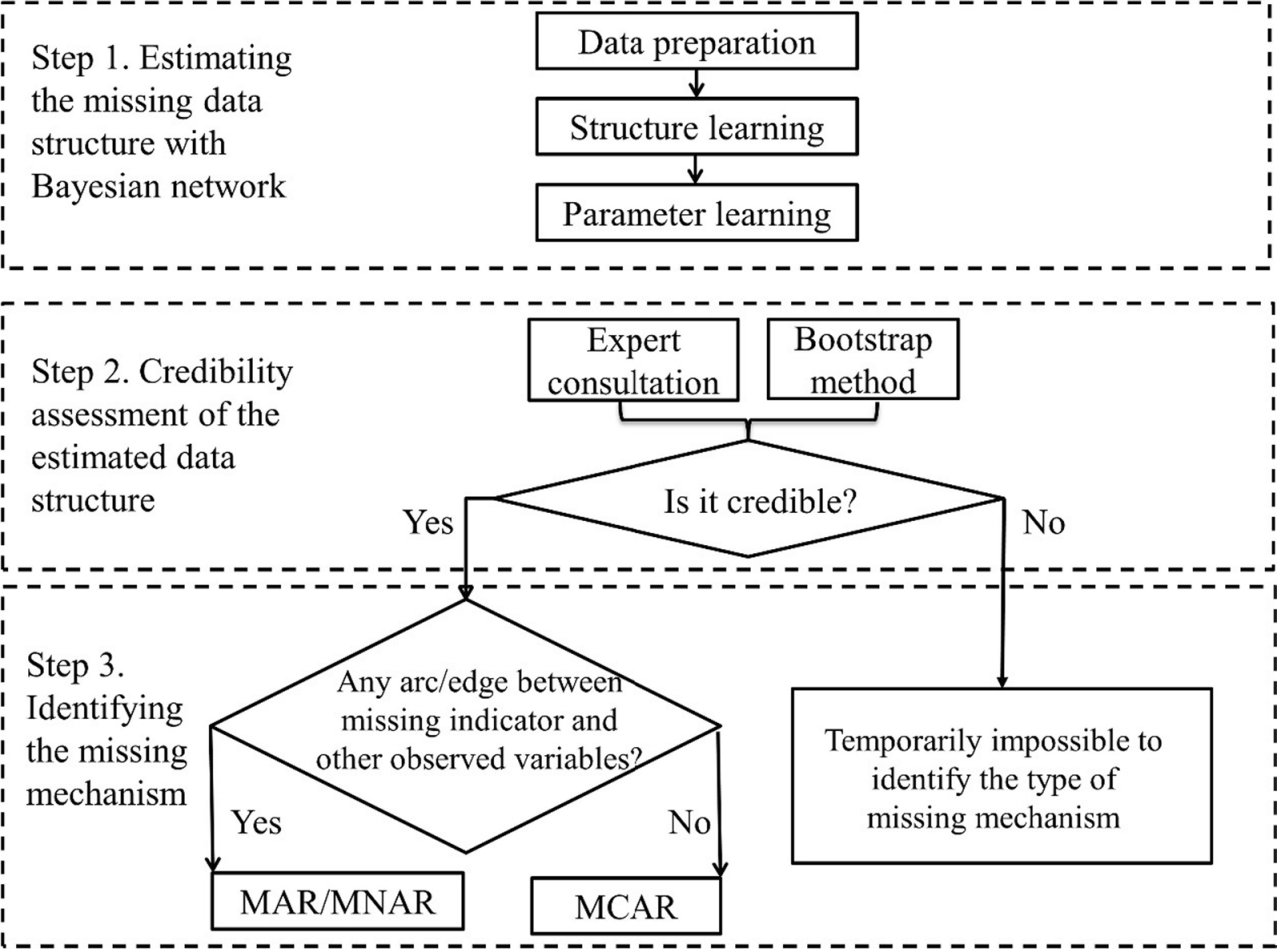
The flowchart of BN-MMI method

The BN-MMI method consists three steps as shown in Fig. 1. Firstly, we estimate the missing data structure by Bayesian network, which will be introduced in "Estimating the missing data structure by Bayesian network" section. Secondly, we will assess the credibility of the estimated missing data structure in "Credibility assessment of the estimated missing data structure" section. Last, according to the estimated missing data structure as well as its credibility assessment results, the missing mechanisms are identified by the criteria to be proposed in "Identify the missing mechanism" section. The details are as follows.

### Estimating the missing data structure by Bayesian network

For a data set with missing value, the task of estimating the missing data structure by Bayesian network could be realized step-by-step through the process of data preparation, structure learning and parameter learning.

#### Data preparation

Prior to the estimation, any variables with missing value should be replaced with its missing indicator. For example, in a study of electronic medical records, the variable *fee* records the medical cost of each patient. However, due to some reasons, the values of *fee* are missing for some patients. Then according to the requirement of data preparation, a new dichotomous variable *missing fee* should be created to indicate whether the *fee* is missing or not for each patient. For the sake of notation convenience, this paper takes the value 0 or 1 for missing indicator, that is, for any given patient, *missing fee* = 0 if his/her value of *fee* is present, and *missing fee* = 1 if his/her value of *fee* is absent. In such way, the data preparation ends up with a newly updated data set, which contains the missing indicator as well as the other variables without missing value. Without special emphasis, all the following implementations will be carried out on the updated data set.

#### Structure learning

Structure learning is to construct the topological structure of the Bayesian network from the data set as well as prior human knowledge and expertise [16]. It corresponds to the model selection in classic statistical methods. Once the structure is determined, it could represent the joint probabilistic independence/dependence relations among variables, that is, arcs in the graph represent probabilistic conditional dependence, otherwise the absence of arcs represents the probabilistic conditional independence. Several algorithms have been proposed and well verified in the literature for structure learning, and the hill climbing algorithm is employed in this study [20]. The algorithm starts the construction of the topological structure with an empty graph, then it adds, deletes and reverses one arc at one time until the topological structure can no longer be improved. Once this step finish, the independent/dependent relations among all variables (including missing variable) under consideration would be identified.

#### Parameter learning

Parameter learning is to estimate the conditional probability for each node in a given topological structure of the Bayesian network. This task can be performed efficiently by estimating the parameters of the local distributions implied by topological structure, which was obtained from structure learning [16]. More precisely, according to the Markov properties of Bayesian network (i.e., each variable is conditionally independent of its non-descendants given its parent variables) [21], the joint probability distribution of multiple random variables in the global distribution could be factorized as a product of conditional local probability distributions associated with every single variable. For example, in the case of *n*-dimensional discrete random variables $X=\left\{{X}_{1},{X}_{2}, \ldots ,{X}_{n}\right\}$, the factorization of the joint probability distribution is given by:

1 $${P}_{X}(X)={\prod }_{i=1}^{n}{P}_{{X}_{i}}({X}_{i}|{\Pi }_{{X}_{i}}),$$

where ${\Pi }_{{X}_{i}}$ is the set of the parents of ${X}_{i}$ in the topological structure. The factorization process of the continuous random variables is similar so that we omit the details here. In addition, the normal distribution is specified for quantitative variables and binomial or multinomial distribution is specified for qualitative variables, respectively. Finally, the Bayesian parameter estimation method is used in this study for parameter learning [20].

### Credibility assessment of the estimated missing data structure

Once the missing data structure is estimated by the Bayesian network, it is quite necessary to assess whether the estimated missing data structure is credible before moving to further steps, otherwise the identification results would not be so convincing. However, since we would never know the true underlying missing data structure in practice, two alternative ways are recommended. The first way is to consult the experts from the corresponding field. Since the experts accumulate a large amount of knowledge and experience and would probably be familiar with the reasons for data missing, they are just in the right position to assess the credibility of the estimated missing data structure by comparing it with reality. In addition, the expert consultation could be easily carried out in practice by some highly advanced interviewing techniques such as the Delphi method [22]. The other way of credibility assessment is through the bootstrap method. Specifically, the method begins by resampling the original data which contains missing elements with replacement by *N* times, which creates *N* replications of the original data set. Then Step 1 is applied to both the original data set and the *N* replications, ending up with one estimated missing data structure from the original data set as well as the other *N* estimated missing data structures from the replications. Theoretically, if the estimated missing data structure from the original data set could reveal the relations among variables robustly, then it is rational to believe it is not that much different from the other *N* estimated missing data structures from the replications; otherwise, it is plausible to deny the credibility of the estimated missing data structure due to its poor repeatability. In this study, we set the credibility threshold for the bootstrap method to be 90%, which means for each relation a given pair of variables is judged to be credible by the bootstrap method if only it could be verified in more than 90% of the replications. We choose both the expert consultation method and the bootstrap method to combine practical experience and data information, and to make the results more credible.

### Identify the missing mechanism

There are two situations in identification criteria according to the result of credibility assessment in the last step.

In the first situation, if the estimated missing data structure passes through the credibility assessment in Step 2, then it is plausible to assume that the estimated missing data structure could reliably characterize the probabilistic dependence/independence relations between missing indicator and other variables. At this point, it is easy to identify the missing mechanism by observing the estimated missing data structure and reviewing the definitions of missing mechanism. Say concretely, the absence of arcs between missing indicator and the other observed variables indicates the MCAR mechanism, while the presence of such arcs refers to the missing mechanism is not MCAR mechanism, but may be MAR or MNAR mechanism.

In the second situation, when the estimated missing data structure is incredible, it is temporarily impossible to identify the type of missing mechanism. Specifically, if the estimated missing data structure is not credible, the probabilistic dependence/independence relations between missing indicator and other variables cannot be reliably characterized by the structure, so the estimated missing data structure cannot be used to identify the type of missing mechanism.

## Simulation study

In order to assess the performance of our method in identifying the missing mechanism, two specific objectives were set in the simulation study. The first was to evaluate the properties of the BN-MMI method itself, and the second was to compare the BN-MMI method with the traditional logistic regression method. To this end, the following process was carried out.

### Simulation design

The simulation design is divided into two steps: first is to build the structure of the simulation model, and the second is to set up the simulation scene and generate simulation data.

#### Structure construction

To make our simulation approximate to the real world clinical trial as much as possible, we included three variables that were of interest for patients and clinicians from the selected hospital: *tumor type* (0, benign tumor; 1, malignant tumor), *operation* (0, without operation; 1, with operation) and *twoweeks* (0, patient left hospital within two weeks; 1, patient left hospital after more than two weeks).

By analyzing the data set selected in the empirical study, we can see that 88% of the patients with benign tumor received operation, while only 58% of the patients with malignant tumor received surgery. In addition, the discharge rate within two weeks of patients receiving operation (52%) was lower than that of patients without operation (63%). After various discussion with clinical experts, the assumed distribution for the data and full specification of the required parameters were set to keep resemble to clinical practice. To be more concrete, we assumed 58% of the tumor patients were diagnosed with malignant tumor while the left was diagnosed with benign tumor. In addition, 89% of the patients with benign tumor received operation, compared with merely 62% of the malignant tumor patients received operation; meanwhile, those patients who received operation had lower discharge rate within two weeks (51%) than those who did not receive operation (65%). Under such scenario, the variable *tumor type*, *operation* and *twoweeks* could be generated by using the binomial random number generators.

$$\begin{gathered} P\left( {tumor \, type = {1}} \right) \, = 0.{58}, \hfill \\ P\left( {operation = {1}|tumor \, type = 0} \right) \, = 0.{89},{\text{ P }}\left( {{\text{operation}} = {1}|tumor \, type = {1}} \right) \, = 0.{62}, \hfill \\ P\left( {twoweeks = 0 \, |operation = {1}} \right) \, = 0.{51},P\left( {twoweeks = 0 \, |operation = 0} \right) \, = 0.{65}. \hfill \\ \end{gathered}$$

On the basis of the generated data set, we introduced another variable *missing fee*, which was a missing indicator for the variable of *fee*, that was, *missing fee* = 1 when the value of *fee* was missing; otherwise, *missing fee* = 0 when the value of *fee* was not missing.

#### Scenario setting and data generation

**Fig. 2:**
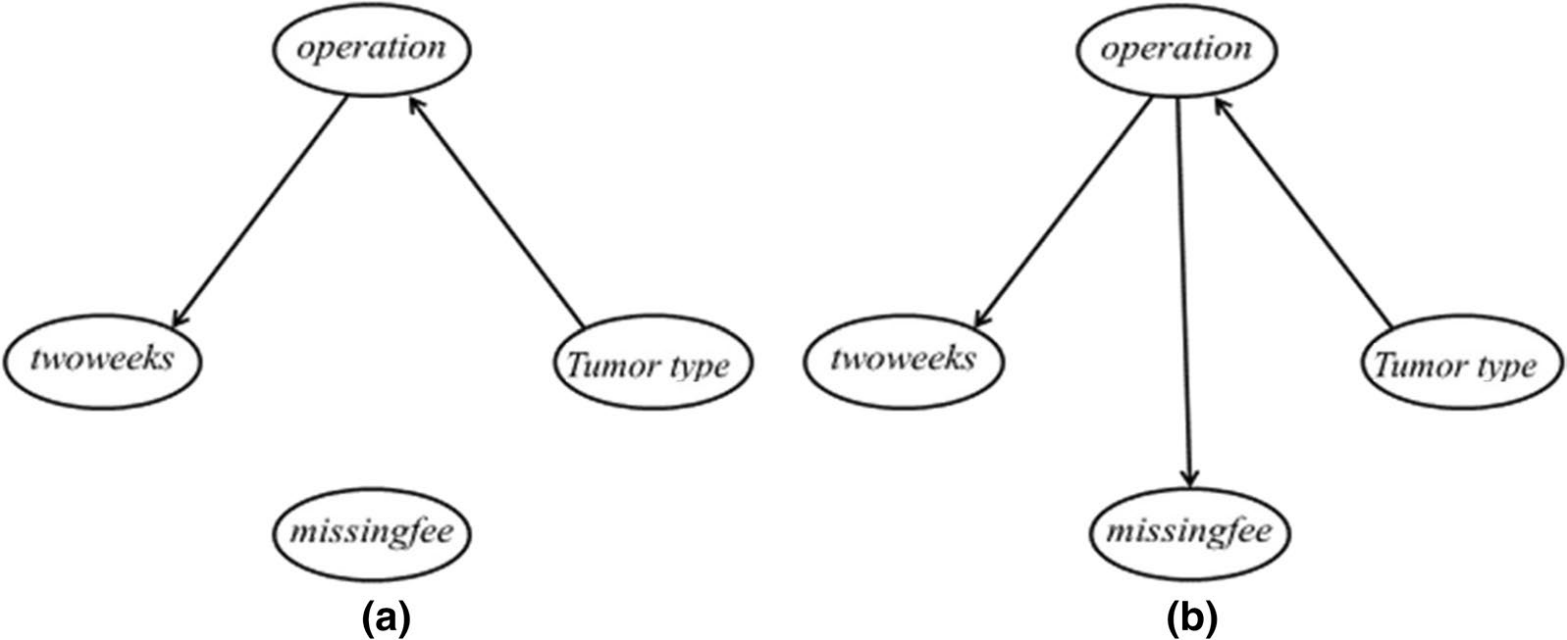
The simulated missing data structure: **a** MCAR; **b** MAR

According to the objective of the study, eight types of simulation scenarios were built, which were the level combinations of missing rate of *fee* (5%, 10%, 15% and 20%) and missing mechanisms (MCAR and MAR). Figure 2 illustrated the missing data structures of these two missing mechanisms, which were created as below.

1. The missing data structure of MCAR was created by generating 0–1 random numbers for *missing fee* with binomial distribution *B* (*n*, *p*), where *n* was the sample size for each time of simulation, and *p* was set to be each of the predefined missing rates of *fee*. In this way, *missing fee* had nothing to do with the rest of variables (Fig. 2a). Since the sample size for per realization was 2000, *n* was 2000. Since we wanted to study the scenarios with missing rates of 5%, 10%, 15% and 20%, *p* were 5%, 10%, 15% and 20% respectively.
2. For the missing data structure of MAR, since *missing fee* was related to *operation* (the reasons would be further explained in the empirical study) and the missing rate of fee for the non-operation group was 2.5 times as high as that for the operation group in practice, it was reasonable to set the missing rate of *fee* for the non-operation group (*p*_1_) to be 2.5 times as high as that for the operation group (*p*_2_). Thus given the overall missing rate of *fee*, we could separately calculate *p*_1_ and *p*_2_. Then the 0–1 random numbers for *missing fee* of either group would be generated, respectively. As a consequence, there was an arc between *missing fee* and *operation* under such a circumstance as shown in Fig. 2b.

For each simulation scenario, 10,000 realizations with a sample size of 2000 per realization were generated. For each simulated data set, the BN-MMI method was applied in the way as described in part 2. All the simulation and analysis work was done in R 3.2.3(the R Foundation for Statistical Computing), a free software environment for statistical computing and graphics. Computing Packages {*bnlearn*} and {*MissMech*} were downloaded from the Comprehensive R Archive Network (CRAN) at http://cran.r-project.org/ and installed in advance.

### Performance measures

In order to achieve above objectives, we evaluated the estimated missing data structure by three kinds of performance measures: *validity*, *simulation consistency* and *robustness*. The *validity* considered the coincidence of the overall underlying simulated missing data structure (the real data structure) and the estimated one obtained by the BN-MMI method. Since we would never know the true underlying missing data structure in practice, we used two alternative ways to determine the real data structure, one was expert consultation, and the other way was bootstrap method. Therefore, the overall underlying simulated missing data structure is the real data structure. Similarly, the *simulation consistency* also considered coincidence, but it only concentrated on the pairwise level (i.e., the relation of two variables) rather than the overall structure level. It was called *simulation consistency* to differ from the *real-study consistency* which would be introduced in the empirical study. For example, for any two variables A and B, if 9116 out of 10,000 simulations identify the true relationship between variables A and B, the *simulation consistency* is 9116/10,000 = 91.16%. In addition, since *validity* would be repeatedly calculated in different simulation scenarios, we took the *robustness* as a measurement to reflect the variation of *validity* across different scenarios, which represents the variation degree of *validity* under different missing ratio of corresponding missing mechanism.

The formulas of all performance measurements in the simulation study were given in Table 1. It should be noted that although we used the *validity* measure to compare the BN-MMI and logistic regression method, there was slightly different meaning for either of them. As shown in Table 1, the *validity* of the BN-MMI method indicated the truth of results for the overall missing data structure. However, since the logistic regression method only concerned about the regression coefficients of independent variables on the missing indicator, the meaning of *validity* for logistic regression method was completely restricted to the truth of the relations between missing indicator and the other variables. In other words, the definition of *validity* for the BN-MMI method was much stricter than that of the logistic regression method. However, if the BN-MMI method could outperform the logistic regression method under this circumstance, such difference would not jeopardize the comparison results, but rather indicate the superiority of BN-MMI method from a strict point of view.

**Table 1:** Performance measures and their formulas used in the simulation study

Performance measure	Formula
*Validity* of the BN-MMI method	(the number of times when the estimated missing data structure completely coincides with the real one)/(the total number of simulations)
*Validity* of the logistic regression method	(the number of times when the estimated missing data structure coincides with the real one in terms of the relations between missing indicator variable and other variables)/ (the total number of simulations)
*Simulation consistency* for the relation between variable A and B	(the number of times when the estimated relation between variable A and B is true) /(the total number of simulations)
*Robustness*	$\frac{{\sum }_{i=1}^{N}{\left(validit{y}_{i}-\overline{validity}\right)}^{2}}{\left(N-1\right)}$, where *N* is the total number of simulations, and *i* refers to the *i*-th simulation

### Simulation results

According to the two aforementioned objectives for the simulation study, the results were summarized in Tables 2 and 3 respectively.

**Table 2:** The validity, simulation consistency and robustness of the BN-MMI method

Missing mechanism	Missing rate	*Validity*	*Robustness*	simulation consistency*									
				*T* → *M*	*M* → *T*	*T* → *O*	*O* → *W*	*W* → *O*	*O* → *M*	*M* → *O*	*W* → *M*	*W* → *T*	*T* → *W*
MCAR	0.05	**0.9698**	0.0018**	*0.0069*	*0.0000*	**0.9931**	**0.9838**	*0.0080*	*0.0058*	*0.0003*	*0.0065*	*0.0006*	*0.0000*
	0.10	**0.9727**		*0.0057*	*0.0000*	**0.9943**	**0.9847**	*0.0074*	*0.0052*	*0.0005*	*0.0053*	*0.0003*	*0.0000*
	0.15	**0.9686**		*0.0062*	*0.0000*	**0.9938**	**0.9829**	*0.0082*	*0.0059*	*0.0003*	*0.0075*	*0.0000*	*0.0000*
	0.20	**0.9697**		*0.0060*	*0.0000*	**0.9940**	**0.9831**	*0.0079*	*0.0074*	*0.0003*	*0.0067*	*0.0003*	*0.0000*
MAR	0.05	**0.9334**	*0.0235*	*0.0030*	*0.0000*	**0.9970**	**0.9889**	*0.0082*	0.9451	*0.0077*	*0.0011*	*0.0000*	*0.0000*
	0.10	**0.9794**		*0.0003*	*0.0000*	**0.9997**	**0.9889**	*0.0071*	0.9911	*0.0086*	*0.0004*	*0.0002*	*0.0000*
	0.15	**0.9790**		*0.0004*	*0.0000*	**0.9996**	**0.9874**	*0.0084*	0.9913	*0.0087*	*0.0001*	*0.0002*	*0.0000*
	0.20	**0.9825**		*0.0004*	*0.0000*	**0.9996**	**0.9882**	*0.0092*	0.9952	*0.0048*	*0.0004*	*0.0004*	*0.0000*

^*^For the sake of convince, let *T* stand for *tumor type*, *M* for *missing fee*, *O* for *operation*, and *W* for *twoweeks*

^**^For the performance measures, the numbers in italic font suggested the lower they were, the better the performance was; on the contrary, the numbers in underlined bold font suggested that the higher they were, the better the performance was. Italics and underlined bold here were marked by the author, which  only for ease of reading and have no special meaning

**Table 3:** The *validity* of BN-MMI and logistic regression method

Missing mechanism	Missing rate	BN-MMI	Logistic regression
MCAR	0.05	**0.9698**	0.9476
	0.10	**0.9727**	0.9493
	0.15	**0.9686**	0.9469
	0.20	**0.9697**	0.9502
MAR	0.05	0.9334	**0.9479**
	0.10	**0.9794**	0.9475
	0.15	**0.9790**	0.9493
	0.20	**0.9825**	0.9513

Table 2 provides the *validity*, *simulation consistency* and *robustness* results of the BN-MMI method in different simulation scenarios. The detailed meanings of the three indicators please see in "Performance measures" section. The *validity*, *simulation consistency* and *robustness* of the BN-MMI method were good under both the MCAR and MAR missing mechanisms. In Table 2 the *validity* of these two missing mechanisms yielded the range from 0.9334 to 0.9825, which indicated that the estimated missing data structures by the BN-MMI method were approximately coincided with the real ones. Meanwhile, the close-to-zero *robustness* just revealed very small variational degree of *validity*, which confirmed the robust performance of the BN-MMI method during each simulation under the MCAR and MAR mechanisms. Furthermore, the *simulation consistency* was approximately near either 1 or 0, conditioned on whether its corresponding true relation existed or not. It should also be noted that under either the MCAR or MAR mechanism, both the *validity* and *simulation consistency* did not change dramatically with different missing rates.

Table 3 provided the comparison results between the BN-MMI and logistic regression method. It could be seen that BN-MMI had good performance under both MCAR and MAR mechanism. Meanwhile, the *validity* of BN-MMI was higher than that of the logistic regression method on average. In addition, considering the *validity* of BN-MMI was stricter than that of the logistic regression method, it was more convincible to conclude the outperformance of BN-MMI method in contrast to the logistic regression method in identifying missing mechanisms.

## Empirical study

We carried out a study on real data example of medical records collected from a hospital. Since the hospital was one of the first-class hospitals in China, it was plausible to believe that the example was representative and illustrative. The research data came from the medical record department of the hospital. There were 1038 records of patients who visited the hospital during the year of 2016, which included 7 variables such as *tumor type, operation, twoweeks, missing fee, transfer, sex* and *age60*. The definitions of aforementioned variables *tumor type*, *operation*, *twoweeks* and *missing fee* given previously. Besides, the medical records also contained following variables: (1) *transfer*, indicating whether the tumor had transferred (*transfer* = 1) or not (*transfer* = 0); (2) *sex*, where *sex* = 1 for male and *sex* = 0 for female; (3) *age*60, recording whether the patient was older than 60 (*age*60 = 1) or not (*age*60 = 0). The structure of the missing data was shown in Table 4, where ○ indicated data was observed and × indicated missing. The column on the far right of the figure showed the total number of cases on the left, and the row at the bottom of the figure showed the number of missing for about each variable. For example, the first row indicated that all variables were observed and there were 885 such cases in total; In the second row, only the variable of *fee* had missing values while there were 153 such cases. Tumor type, operation, twoweek, transfer, sex and age60 were no missing values, while *fee* had 153 missing values and the missing rate was 14.74%. Data description was carried out before analysis. However, due to the length limitation of this paper, Tables 5, 6 and 7 just listed some important relations found in the data.

**Table 4:** The structure of the missing data in selected hospital

*Tumor type*	*Operation*	*Twoweek*	*Transfer*	*Sex*	*Age60*	*Fee*	Sum
○	○	○	○	○	○	○	885
○	○	○	○	○	○	×	153
0	0	0	0	0	0	153

**Table 5:** The relation between *operation* and *missing fee*

	*Missing fee* = 0	*Missing fee* = 1	${\chi }^{2}$ statistics	p-value
*Operation* = 0	235 (75.32%)	77 (24.68%)	33.95	< 0.0001
*Operation* = 1	650 (89.53%)	76 (10.47%)

**Table 6:** The relation between *tumor type* and *operation*

	*Operation* = 0	*Operation* = 1	${\chi }^{2}$ statistics	p-value
*Tumor type* = 0	51 (12.13%)	366 (87.77%)	103.96	< 0.0001
*Tumor type* = 1	261 (42.03%)	360 (57.97%)

**Table 7:** The relation between *operation* and *twoweeks*

	*Twoweeks* = 0	*Twoweeks* = 1	${\chi }^{2}$ statistics	p-value
*Operation* = 0	198 (63.46%)	114 (36.54%)	11.29	< 0.0001
*Operation* = 1	377 (51.93%)	349 (48.07%)		

**Fig. 3:**
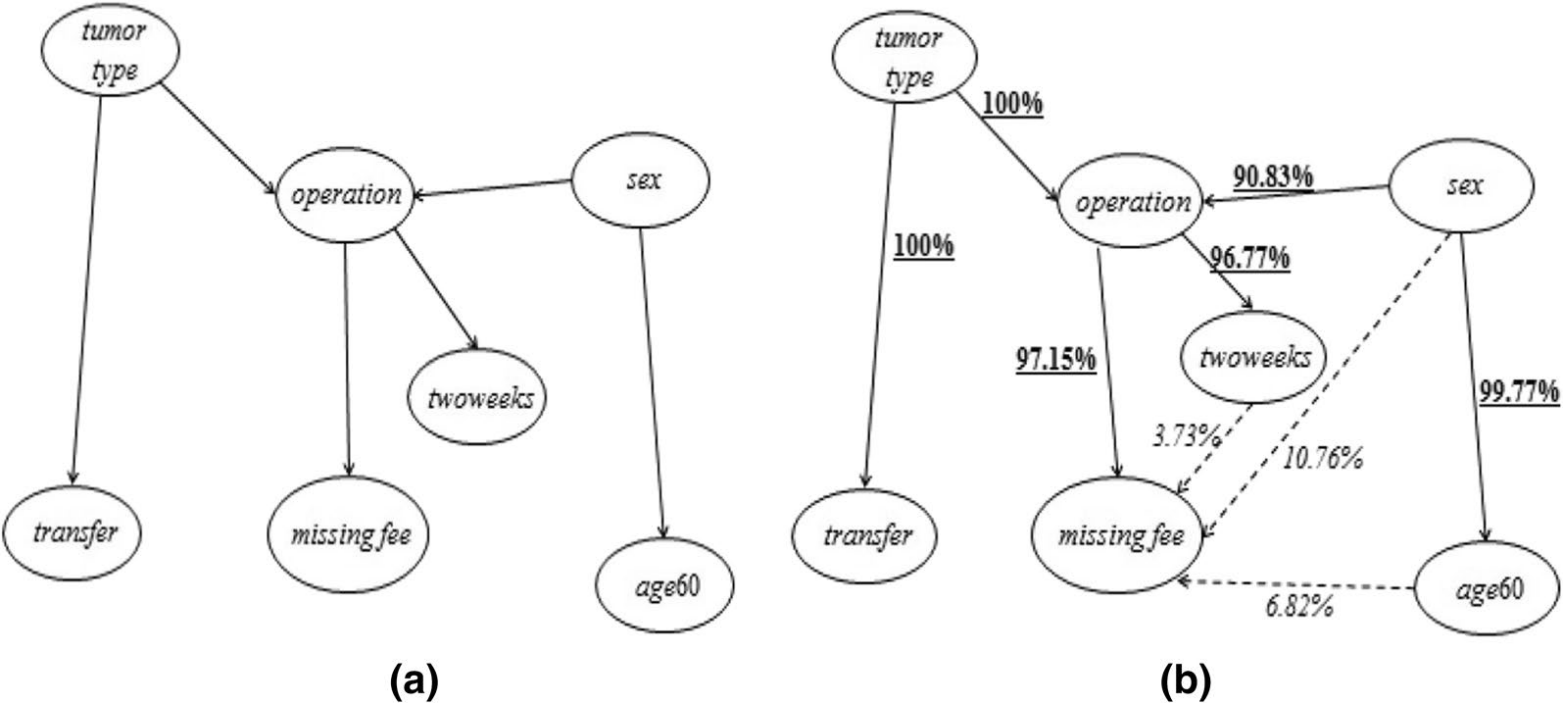
The BN-MMI method applied to real data example. **a** The missing data structure estimated by the clinician expertise; **b** the missing data structure estimated by the BN-MMI method. In **b**, the solid line indicated the two estimating approaches were consistent, where the underlined bolded numbers represented the real-study consistency; the dashed line indicated the two estimating approaches were inconsistent, where the italic numbers represented the real-study inconsistency

Besides, prior to the application of BN-MMI method, we also asked some experienced clinicians to estimate the missing data structure according to their knowledge and experience, as shown in Fig. 3a. Specifically, the clinicians thought the missing rate of *fee* was higher for the non-operation group than that for the operation group, and their reasons were summarized below.

1. The hospital required the patients who received operation pay their fees in advance, while this was not necessary for those who did not receive operation. Therefore, the fee of the operation group was less likely to be missing than that of the non-operation group.
2. According to the workflow of the hospital, the fee data was first entered into the financial database and then copied to the medical record database. Since the two databases were separated there were usually two ways to copy the fee data. One way was to copy by computer automatically. But due to some administrative and technical reasons, it usually took a longer time to copy the fee data of non-operation group into the medical record database than that of the operation group, and the longer time could just lead to the higher risk for the missing fee data. The other way was to copy the fee data manually, which was usually done by nurses and interns. However, due to their heavy workloads, some of them decided to give priority to copying the fee of operation group because they thought the fee of the non-operation group was not so important. Such personal decision could again interpret the reasons for unbalanced missing rates of fee between operation and non-operation groups.

Next, the BN-MMI method was applied to estimate the missing data structure. Specifically, the bootstrap method was used to repeat the application of BN-MMI method for 10,000 times, which resulted in 10,000 estimated missing data structures by BN-MMI method. We then compared those 10,000 data structures with the one previously estimated by expertise. The comparison results were summarized by the measure of *real-study consistency* and *real-study inconsistency*, where for a given variable A and B, *real-study consistency* = (the times when the relation of A and B estimated by BN-MMI method and expertise were consistent)/10000 × 100%, and *real-study inconsistency* = (the times when the relations of A and B estimated by BN-MMI method and expertise were inconsistent)/10000 × 100%.

Figure 3b presented the missing data structure estimated by the BN-MMI method as well as the results of *real-study consistency* and *real-study inconsistency*. It could be concluded that the *real-study consistencies* were relatively high while the *real-study inconsistencies* were relatively low. This indicated that the results of BN-MMI method were in high accordance with expertise, therefore the estimated missing data structure by the BN-MMI method indeed made practical sense. Besides, considering the *real-study consistency* of {*operation* → *missing fee*} was as high as 97.15%, it was plausible to conclude that the missing mechanism of the real data example was not MCAR mechanism, but may be MAR or MNAR mechanism.

## Discussion

This paper explores the application of Bayesian network to the identification of missing mechanisms for the first time and proposes the BN-MMI method. Besides the promising features of Bayesian network which have already been proved by previous studies, this paper further carried out both simulation and empirical studies to verify the properties of *validity*, *consistency* and *robustness* of Bayesian network, in terms of missing mechanism identification. Meanwhile, the simulation study showed that the *validity* of BN-MMI was higher than that of the logistic regression method on average. These evidence not only prove the BN-MMI method itself to be efficient, reliable and applicable, but also shed light upon the potential application of BN-MMI method to a broader range of missing data issues in medical studies, such as the investigation of missing values, dealing with missing data and deep-learning of missing data structure with latent variables [23], etc. There is no doubt that the latter is even more important since all these missing data issues are becoming more common and complex in medical research with the advent of the big-data era [24].It is plausible to anticipate that through step-by-step efforts, our present work will contribute to further development of missing data analysis in the future.

We adopt the common belief that “all models are wrong, but some are useful”, so it is necessary to pay attention to the suitable conditions for the model. As for the BN-MMI method, it can identify the MCAR mechanism effectively, but it’s impossible to further distinguish MAR and MNAR mechanism. This limitation is universal: according to little and Rubin [7], it is difficult to identify the MNAR mechanism because the real value of the missing value is unknown. However, our method at least requires credibility assessment of the estimated missing data structure (i.e., Step 2 of the method) before identifying missing mechanism (i.e., Step 3 of method). In such a way human knowledge and expertise could be used to guarantee proper use of BN-MMI method as much as possible.

Considering this is a preliminary study of the application of Bayesian network to missing mechanism identification, this paper puts main focus on the issue of univariate missingness. Although some interesting findings have been made in this study, there are wide area to study, such as the missing mechanism identification in the case of multivariate missingness and/or continuous variables [25]. There are two advantages in the extension from single variable to multi variable using Bayesian network to identify missing mechanism. The first is the convenience of variable setting: using BN-MMI method to identify data missing mechanism is to set the missing variables as "missing indicator variables", and BN-MMI method can easily set multiple "missing indicator variables" at the same time and build Bayesian model. The second is the convenience of revealing the relationship: the Bayesian model constructed is a network structure, which can pay attention to multiple variables and reveal the relationship among multiple variables at the same time. The above two characteristics of Bayesian method lay a good theoretical foundation for its multivariable research. Therefore, Bayesian network has a wide range of research fields and great application value in missing mechanism identification, and using Bayesian network to identify the data missing mechanism under multivariable will be our next research direction.

## Data Availability

The simulation study data can be generated according to the scenario setting and data generation in "Scenario setting and data generation" section, and the empirical study data used to illustrate the applicability of BN-MMI method is available from the corresponding author upon request.
